# "The Hospital" Nursing Mirror

**Published:** 1901-01-19

**Authors:** 


### "The Hospital" Nursing Mirror.

Being the Nursing Section of "The Hospital."

[Contributions for this Section of "The Hospital" should be addressed to the Editor, "The Hospital" Nursing Mirror, 28 & 29 Southampton Street, Strand, London, W.C.]

### Notes on News from the Nursing World.

### DISPATCH OF MORE NURSES TO SOUTH AFRICA.

The Secretary of State for War informs us that thirty nursing sisters of the Army Nursing Service Reserve are proceeding to South Africa. Some of them left in the *Avoca* on Monday, and the remainder leave in the *Canada* on Saturday. The names of the nurses are as follows:—A. R. Allaway, E. M. Bailey, M. G. Barsdorf, K. Beaufoy, C. A. Calladine, C. T. Collins, A. E. Davies, E. M. Fisher, J. Grewer, C. Harris, E. B. Hill, C. Hodgkin, A. S. Johnston, E. Jones, A. Luscombe, C. Macpherson, G. E. Mall, L. E. Mitchell, M. Morrison, J. M. Mortlock, E. Newbold, C. Nolan, J. Orr, S. E. Richmond, C. M. C. Rogers, F. E. Scott, M. J. Spencer, E. L. Warren, D. E. J. Wetton, and E. Wilcock. Miss Agnes E. Davies was trained at Cromwell House Convalescent Home, Highgate. Subsequently, she was staff nurse and head nurse at Lincoln County Hospital, and since 1896 has been engaged in private nursing. Miss Frances Ellinor Scott was trained at the Sussex County Hospital, and has been attached to the Nurses' Co-operation, New Cavendish Street, since 1891. Sister M. Stevenson also sailed in the *Avoca.* She was trained at Mill Road Infirmary, Liverpool, and has since been charge nurse at the South Eastern Fever Hospital, and on the private staff at 123 New Bond Street until last August, when she was sent to Norwich Military Hospital.

### THE PRINCESS OF WALES AND THE ALEXANDRA NURSES.

There are many persons who will respond to the appeal of the Princess of Wales on behalf of the Soldiers and Sailors' Families' Association all the more freely because they know that there is a nursing branch of the organisation in which the Princess takes special interest. Its operations are described in another column, and it will be learnt with satisfaction that the proceeds of the fête held at Chelsea Hospital in 1897 were sufficient to provide the amount needed for the erection of a nursing home at the Curragh Camp, which, by special request, is to be called the "Alexandra." A satisfactory point is that the services of the Alexandra Nurse in Natal, who frequently contributes accounts of her experiences to "Thu Hospital" *Nursing Mirror,* were utilised during the stress of the early stages of the war for the benefit of the sick and wounded soldiers whose families had been sent home.

### THE NURSES OF THE IMPERIAL YEOMANRY HOSPITALS.

Several changes have been made in the nursing staff of the Imperial Yeomanry Hospitals. Sisters Cheetham and MacDonald have been transferred to the P.M.O. Army. They have proceeded to Pretoria, and are doing duty with No. 2 General Hospital. Sister Tillott has been invalided to England. Sister Barnes has gone to Johannesburg for duty in the South African Constabulary Hospital.

### THE DEARTH OF DRESS MATERIAL AT PRETORIA.

An appeal is made by Sir Edmund Verney for funds in order to forward a chest of dress materials to the nurses in the Imperial Yeomanry Hospital at Pretoria. Sir Edmund quotes in support of his appeal a letter from a correspondent, who says:

> The soldiers and officers have canteens and stores where they can buy everything reasonably, but we have to go to ordinary shops, where the prices are exorbitant. We all want new dresses, and can't get the material without sending to England. I actually bought some holland blind material the other day for a dress to wear if I go for a holiday, but I shan't be allowed to wear it in the hospital, as it is not the correct colour—grey. Working in tents soon knocks one's things to pieces.

We do not think that the writer of this letter supposed that the public would be asked to provide the money for new dresses for Army nurses. There is no reason why the friends of the nurses should not, if they like, dispatch to them a nice little present in the shape of dress material; but the sending of the hat round to outsiders in such a case is quite another matter.

### THE "HOSPITAL" CONVALESCENT FUND.

The Hon. Sec. of the "Hospital" Convalescent Fund has received 10s. from Nurse M. E. M., Eastbourne, which she sends "as a thankoffering," and 2s. 6d. from "A Friend." We take this opportunity of saying that the kindly spirit evinced by these spontaneous gifts from nurses to their less fortunate fellow-workers is a source of great pleasure to all connected with The Hospital Convalescent Fund, who know the value to the convalescent of the help which they are thus able to render. At the present moment they have before them the case of a nurse just starting on her career who has broken down in health, and consequently been compelled to give up her profession until she regains her strength. She cannot afford a change of air, which is most necessary to her; but fortunately we are able to assist her in this respect and thus put her in the way of being in a position to again take up the work which she likes and for which she appears to be very suitable.

### THE TRAINING OF WORKHOUSE NURSES.

An important conference of Yorkshire Poor Law representatives has been held at Leeds, under the auspices of the Leeds Board of Guardians. The subject of discussion was the best means of securing a proper and uniform standard of training for nurses in workhouse infirmaries. A resolution in favour of a general uniform standard of training and examination for workhouse nurses in Yorkshire was unanimously adopted. One of the speakers, however, urged that a national, rather than a county, standard should be set up, which elicited from Dr. Hawkyard, of Hunslet, the remark that "if Yorkshire waited for the whole country to take up the question they would wait until Doomsday." We do not share the pessimism of Dr. Hawkyard, for if each county will exert itself as Yorkshire is doing to improve the standard of training, a national standard is far from being a vain dream. But in existing circumstances, the decision arrived at by the conference was prudent. Of course, there is no room for doubt as to what the standard of training of workhouse nurses should be. Dr. McCandlish told the conference that the system of training in the Leeds Workhouse Infirmary is similar to that of a general hospital except in regard to special surgical cases. No lower standard than this can be considered satisfactory. As to two separate standards, which Dr. Hawkyard proposed, and Dr. McCandlish deprecated, its adoption would be injurious to the profession. There will pro bably for some time be nurses who have been insufficiently trained as well as nurses who have been adequately trained; but to formally set up two standards and two grades would be a retrograde step. The conference subsequently nominated a sub-committee to consider the best method of carrying the first resolution into effect, including the superintendent nurses at Leeds, Hull, and Wakefield Infirmaries. Mr. Winter, who pleaded for an examination regulated by the Yorkshire College, said that some of the candidates for the appointment of nurse who came before the Hull Board of Guardians could not write and spell their own names. This is a sufficient reason why their services should be declined; but it is not an argument for an educational as against a professional test.

### NORTH DEVON INFIRMARY, BARNSTAPLE.

The cause of the dismissal of the probationers at the North Devon Infirmary was, we understand, that they refused to sign a new printed code of rules, different, in many points from those set forth in the written agreement which they had previously agreed to sign. No copy of the written agreement seems available, but the printed code appears at any rate to have been either drawn up in a hurry or by an illiterate person. For instance, Rule 8 says: "Sitting and meal rooms lights to be out and the door locked at 10 p.m. All bedrooms lights must be out at 10.30 p.m." To Rule 2, which reads, "Any complaint is to be made in writing to the secretary, who will (at the earliest opportunity) bring it before the proper authorities," is strangely added, "Always wear uniform on duty." Rule 19 details the nature of the practical instructions to be given by the charge nurses to the probationers. The former are enjoined to explain "the details of preparing patients and patients skin for operation," and to instruct in the careful observation of the sick with regard to "delerium." Rule 3 is very oddly worded. It is to the effect that "nurses and probationers shall strictly obey the orders of the medical officers—the house-surgeon and the matron." Is the matron, then, a medical officer? According to our information, she is even untrained. The charge nurses, it seems, never had any rules given to them before they accepted appointments; but when the probationers, who were promised them, were taken away to relieve other wards, and sent off duty without consulting them, they complained. The result was the new code of rules which they consider only make matters worse. They also complained that the wards were left entirely two hours every day for meals, without even a ward-maid in attendance; but the reply of one of the officials was that a convalescent patient was quite as good as a charge nurse to be left with a dying patient. One general and natural objection to the new code is that nothing is said about lectures or teaching of any kind. Possibly the authorities may be able to make an effective reply to the strictures which their action, or inaction, has provoked. But, although in reply to a telegram sent from Barnstaple on January 1st asking us to wire the name of the writer of the letter signed "Onlooker," drawing attention to the North Devon Infirmary, we replied that although we declined to give the name we would insert comment on our correspondent's statements, none has so far reached us.

### LORD LINDLEY AND NURSES' AGREEMENTS.

At the annual meeting of the Norfolk District and Cottage Nursing Association, the Countess of Leicester in the chair, Lord Lindley mentioned that the Norfolk County Council had voted £50 to the funds of the Association. We are glad to find that Lord Lindley is taking more than a conventional interest in the progress of this excellent organisation. In his speech as chairman of the executive, he mentioned that he had lately had the opportunity of seeing a number of the agreements which had been entered into with the nurses by the Association. "Some of them," he continued, "were very strange-looking documents, and so unbusinesslike were a few of them that it occurred to him that it would be desirable if a form of agreement were prepared by a person of competent skill, and that there should be some rules for the guidance of the nurses printed on the back." Criticism of this sort is easy, but Lord Lindley did not leave off here. He volunteered to place his great professional skill at the service of the Association, and to prepare a suitable document. This is real practical help. Next to the observance of care in the selection of nurses, nothing is more important than the terms of the agreements entered into. Many of the troubles which agitate the nursing world are due to the vagueness of agreements, and clearness is at least as requisite in the interests of the nurse as of her employer. Lord Lindley, who has in the person of Dr. Burton-Fanning an exceptionally able colleague, must be congratulated not only upon having discovered the weak spot in the armour of the Norfolk Association, but also upon closing it up.

### THE MALDON NURSING ASSOCIATION.

The upshot of the controversy which has agitated members of the Maldon Nursing Association for some weeks is that the committee resigned last week in a body, and the nurse to whom they gave permission to refuse to attend patients under the care of the doctor whose treatment she did not approve resigned with them. This is in a sense satisfactory, since a new committee has been elected who, it may be assumed, will not give any countenance to the pernicious view that it is within the province of a nurse to criticise the treatment of a case by the medical man under whom she is working. But it is a pity that the Maldon ladies should have taken umbrage, or been instigated to take it, in a matter in which they were obviously in the wrong; and their wholesale resignation may, as a speaker at the last meeting pointed out, possibly injure the Association. Some very excellent advice was given them by the original starter of the organisation, Mrs. Scott of Witham, who is naturally interested in its well-being, and wrote as follows:—

I think most emphatically that it is no part of a nurse's duty to question the treatment of any doctor under whom she may be called upon to work. Her one and only duty is to carry out the doctor's orders in an efficient manner. The patient or his relatives decide which doctor is to attend, and, presumably, calls in that doctor in whom he or they have most confidence. It is in my opinion absolutely unjustifiable for the nurse to say or do anything to undermine the patient's confidence in his medical attendant. I consider that the Committee should be most careful not to do or allow to be done anything likely to cause ill-feeling between themselves and the various doctors whose patients their nurse is attending. They are not the judges as to the propriety or impropriety of a doctor's treatment of his patients, and if their nurse refuses to work impartially under any doctor whose case she may be called upon to attend, there are two courses open—either that a nurse should be found who will perform her duties or the Association should withdraw their subscriptions.

This advice was all the more entitled to attention because Mrs. Scott, before her marriage to a medical man, was a trained nurse attached to the private staff at the London Hospital. But it did not convince the Committee that they had made a mistake, and as they were not able to see things in this light, the course they pursued became practically inevitable. We can only hope that the sick poor of Maldon will not be in any way sufferers from the unfortunate controversy which has disturbed the peace of the town.

### THE REPORT OF A WORKHOUSE INFIRMARY SUPERINTENDENT.

Whether, as alleged by one of the Exeter Corporation of the Poor, the superintendent nurse at the workhouse hospital was "moved" to write to the Local Government Board by a guardian, or whether on her own account she reported a case of gangrene which had been the cause of great offensiveness to one of the men's wards, and complained of the stench from the lavatories, she has been the means of emphasising an intolerable condition of things. There has been a long discussion on the subject by the Exeter Guardians, but although Mr. Preston Thomas, the Local Government Board inspector, was present and reiterated the statement he had already made, that the workhouse infirmary accommodation at Exeter is deplorable, while several guardians denounced the sanitary, or insanitary, arrangements, a dilatory amendment to the motion recommending the erection of a new hospital with a lying-in ward and accommodation for the nursing staff, was adopted. At present, therefore, the matter remains unsettled, but the superintendent nurse has done her duty, and in the end the reformers will have their way.

### A CRY FROM FAR ACHILL.

We do not understand why the Congested Districts Board which sent a nurse to Achill, Ireland, now says it can no longer maintain her. The need for her presence is admittedly just the same as it was, and the work she is doing is unquestionably good. In the year before she went to Achill there were 24 deaths in childbed, but since she has been there scarcely any accidents have occurred. She is one of the Queen's Jubilee Nurses, and the committee of the Institute are asking for contributions to raise an annual £120 to keep her where she is, the island itself being too poor to conform to the rule of paying a portion of the cost of the nurses. We hope that they will get it, sooner than that the poor folks at Achill should be deprived of the services of a nurse; but the Congested Districts Board must be in a sad state of penury.

### FIRST AID AND HOME NURSING.

There is an impression on the part of some people, particularly amongst those who have many friends in the nursing profession, and are consequently jealous for its honour, that instruction received by amateurs in "First Aid" or "Home Nursing" tends to encourage the idea of relatives with a little knowledge being able to do the work of a trained nurse. That this impression is erroneous was demonstrated, not for the first time, on Tuesday afternoon, when a course of lectures by Dr. James Cantlie was commenced at the Polytechnic, Regent Street. The lecturer explained that First Aid was a perfectly distinct and separate branch of surgery from that practised by either a doctor or a nurse. Doctors and nurses did not learn it in their ordinary hospital training. A First Aid student must not therefore imagine that the knowledge acquired in these lectures in any way overlapped professional knowledge; the aim of the course was to give such instruction as would enable them to do the best that was possible in an emergency until professional help arrived, and to make the best use of any instrument that was to be had at the moment; if no splint was near, one must be made from an umbrella or a walking stick; and a bandage must be created out of, *e.g.*, a pocket handkerchief or a pair of braces. The instruction would include, not only how to treat the patient, but what to use. Incidentally, much useful information was given in this lecture, and the ultimate advantage both to patient and nurse of such prompt remedies as an amateur can apply being given at once, must be apparent to all.

### THE NURSES' HOSTEL.

The directors of the Nurses' Hostel Company have decided, with the consent of the shareholders, to increase the accommodation by building a similar block on a vacant site opposite the present hostel in Francis Street. The demand quite exceeds the supply, and the directors intend in the proposed block to try to meet the wants of those nurses who would like to rent an unfurnished room. An issue of additional capital of £10,000, in £10 shares, has been determined upon, but before an appeal is made to the general public the new shares to the extent of £8,000 will be offered to the present shareholders.

### HOSPITAL AND STAGE.

At the Vaudeville Theatre, where "Alice in Wonderland" is being performed, there are lantern pictures between the two parts of "Wonderland" and the "Looking-Glass"; some of Sir John Tenniel's original illustrations and Mr. Louis Wain's cats are shown; and last, but not least, there is a photograph of the Lewis Carroll Cot in the Hospital for Sick Children. A little patient occupies it, and a nurse bends over it looking at the child. The pictures are illustrations in the Scrap Book which is being sold for the benefit of the hospital, and may be bought in the theatre.

### SHORT ITEMS.

The Princess of Wales has presented Sir Alfred Cooper, of The Gables, Surbiton, with a diamond pin, accompanied by a letter of congratulation upon the honour of Knighthood conferred upon him by the Queen.—Lectures and classes in preparation for the next examination of the Obstetrical Society of London begin at the Midwives' Institute on Monday next, when the first lecture will be given by Mr. F. R. Humphreys. Nurses who wish to enter themselves for this course should communicate without delay with the Secretary, Trained Nurses' Club, 12 Buckingham Street, Strand, W.C.—At one of the receptions of the Mayor of Burnley this year the nurses of the Union Infirmary of Burnley were most kindly remembered. All of them received an invitation, and about half the staff were able to accept. The nurses went in their uniforms, and thoroughly enjoyed the hospitality extended to them.

### Lectures to Nurses on Medicine and Medical Nursing.

By Norman Dalton, M.D. London, F.R.C.P., Physician to King's College Hospital.

(*Continued from page* 182)

### II.—ON CERTAIN DISEASED STATES: ASTHENIA, PROGRESSIVE ASTHENIA.

There are certain diseased states, such as dropsy, asthenia, fever, &c., which it is important to consider separately, because, although they are not themselves diseases, their presence indicates that the patient is suffering from some definite disease. They are looked upon as "secondary" affections, the disease in the course of which they occur being called the "primary" affection. In the old times many of these secondary affections were considered by physicians to be themselves diseases; for instance, in many old books it is stated that persons suffered from dropsy or died of dropsy, or that certain herbs cured the dropsy. Now, however, we know that dropsy is not itself a disease, but is due, in some cases, to heart disease, in other cases to kidney disease, &c. Nevertheless, dropsy is itself a diseased state which is worth considering quite apart from the real diseases which cause it.

Most of the diseased states to which I shall refer are recognised, not by the presence of one symptom, but by the presence of a collection or group of symptoms, and it is by finding out that certain of these symptoms are present in the patient that we know that the diseased state exists. For instance, when the temperature of a patient is higher than 100°, when his pulse is quicker than normal, and other symptoms can be detected, we know that the diseased state called "fever" is present, but we then have to find out what disease is causing the fever. If we discover that the patient has been only ill for thirty-six hours, that his illness began with a sore throat, and that he has a certain kind of red rash on the chest, &c., we know that his fever is due to the disease called "scarlet fever." If, on the other hand, we find that his liver is large and has a soft bulging swelling on it, we know that the fever is caused by an abscess in his liver.

*Asthenia—Progressive Asthenia.*—The word "asthenia" simply means weakness or debility, and as in practically every disease the patient is not so strong as he ought to be, it may safely be said that asthenia occurs in every disease. Using the term as an adjective, we say that the patient is asthenic. It should be noted incidentally that a great many medical terms which begin with an "a" indicate the absence or the loss of some quality or function which a healthy person should possess; for instance, *a*sthenia means loss of strength; *a*menorrhœa, the absence of the menstrual flow; *a*mnesia, the loss of memory, &c. Now, although the explanation of the word "asthenia" is so very simple, there is a very important diseased state called "progressive asthenia," which occurs in several diseases, and which is characterised by the following group of symptoms. First of all the patient finds that he is disinclined for work and even for the amusements to which he has been addicted. He may have been very energetic, but now all exertion is a trouble. He tires easily and is soon "knocked up." In the course of a few weeks or months he is distinctly weaker; he cannot lift any heavy weight, or walk far, and any effort, especially going upstairs, causes shortness of breath, or even brings on an attack of giddiness and fainting. It should be noted that fainting, or syncope, as it is called, is the very acme of weakness, except, of course, when it is due to nervousness or emotion. Later on the patient becomes too weak to go out of doors, then to go downstairs, and then to leave his bed. The temperature may be subnormal. Finally, the mere exertion of turning in bed and more especially any sudden attempt to sit up, brings on distressing shortness of breath and faintness, and in one of such attacks of fainting he may die. In some cases dropsy occurs in the feet, and in some cases death is hastened by an attack of bronchitis or some other acute illness. The above sequence of symptoms indicates the condition called progressive asthenia. It will be noticed that many of the symptoms, especially the shortness of breath on exertion and the dropsy, are signs of heart disease; but this is due to the fact that, while all parts of the body are equally weak, the heart is such a vital organ that its weakness shows itself most prominently. In some cases the heart is not merely weak, but undergoes fatty degeneration.

Progressive asthenia occurs most typically in the following diseases:—The severe forms of anæmia, such as pernicious anæmia and leucocythæmia, Addison's disease, diabetes, cancer (especially cancer of the stomach), and tuberculosis (especially tuberculosis of the intestines and mesenteric glands). It may, of course, occur in other diseases, such as renal disease, hysteria, chronic alcoholism, &c., but in these other more definite symptoms are usually prominent. In fact, it is in pernicious anæmia and in Addison's disease that it is most important to recognise the presence of progressive asthenia, because there may be very little else in the way of symptoms. It occurs, sometimes as a natural event, in very old people, in whom the brain may show its weakness by loss of memory and an inclination to laugh and cry at trifles. In young people the mind is not usually affected, although the capacity for mental work diminishes with the bodily strength. Asthenia occurs in all prolonged fevers such as typhoid, but more rapidly than in what is usually called progressive asthenia. It is interesting to note that during convalescence from a prolonged fever the symptoms are just the reverse of those of progressive asthenia, *i.e.*, the patient regains strength and becomes more capable of doing things every day.

Most patients with progressive asthenia become thin, so that they lose flesh as well as strength. This is the case in diabetes, cancer, and tuberculosis. But in Addison's disease and in both forms of severe anæmia the patient may be quite fat, although so weak that he cannot move without serious shortness of breath or fainting.

Apart from the disease in which it occurs, the nursing of progressive asthenia does not call for special remark. Strengthening food and strengthening medicines of every kind are ordered by the doctor. The patient should never take a large quantity of food at a time, because if the stomach be loaded the blood is attracted in great quantity to that organ, so that the brain becomes comparatively bloodless, and this may cause fainting. Nurses should also (quite apart from meals) keep brandy close at hand, with water ready to mix with it, for if fainting should occur the rapid administration of a tablespoonful of brandy mixed with three tablespoonfuls of water may save life for the time. Also it is particularly necessary to guard against the exposure of the patient to cold; for cold diminishes vitality, and, further, the slightest bronchitis, or even a common cold in the head, may be sufficient to kill the exhausted patient.

As regards the points to be observed by the nurse for her report, it should be remembered that in the severe cases of anæmia and sometimes in other cases in which there is progressive asthenia, hæmorrhages may occur from any part of the body, so that even a little bleeding from the nose or the appearance of small hæmorrhages in the skin are quite important symptoms. The hæmorrhages in the skin may be as small as fleabites (in which case they are called

"petechiæ"), or so large that they may be taken for the black and blue of a bruise. Hæmorrhages in the skin can be distinguished from the spots which form an eruption by the fact that when pressed on by the finger they remain unaltered, while the spots of an eruption, when treated in the same way, disappear for a second or two. The occurrence of vomiting should of course be noted, and nurses should watch for the onset of dropsy in the legs, as will be explained in other lectures. Lastly, it is very important for a nurse to observe carefully what happens in the fainting attacks which are so common in pernicious anæmia and in Addison's disease, because she may be the only person present when they occur, and because untrained persons usually describe them as a fit, which does not enable the doctor to recognise what kind of attack it was. It is important to notice what the patient was doing when the attack occurred. Most often he has sat up suddenly when lying down, or risen suddenly from his chair, or perhaps he was getting out of bed. If he has time to speak, he may say that he feels giddy, or sick, or that he cannot see; but very often he simply rolls over or falls down, and becomes unconscious. His face is pale, and his pulse is either very feeble or it cannot be felt at all. Lastly, it should be particularly noted whether any movements, such as twitching of the face, jerking of the eyeballs, or contortions of the limbs occur; because, if they do, the attack would not be a true fainting attack, but would be a convulsive fit.

All that can be done for the patient during the attack is to stretch the body flat, keeping the head as low as possible, to loosen the clothing if he is fully dressed, and to give him brandy as soon as he can be got to swallow.

*(To be continued.)*

### Nursing in British Central Africa.

BY A CORRESPONDENT IN KOTA KOTA.

WHEN I came to live in Kota Kota in April, 1899, my arrival was looked upon in the light of an important event. There had never been either a doctor or a nurse resident here before—the native population had doctored themselves, and the Europeans—there were only seven of them—had helped each other as best they could. My coming had been noised abroad so that on the very first morning when I stepped out on to the verandah after breakfast I found quite a small crowd of would-be patients seated on the path awaiting me.

### THE FIRST PATIENT.

The first to accost me was a woman who complained of toothache—she wanted me to draw a large upper molar. As Kota Kota had never been a medical station before there were no tooth forceps, but somebody produced a pair of small gas-pliers and asked if they would answer the purpose. I had never drawn a tooth previously but had seen a good deal of it in the Casualty Ward in a London Hospital, so I grappled with it boldly. The patient sat on the ground with her back to me and held me round the waist—in went the pliers, and to my great joy out came the tooth! It was not even broken.

### THE DISPENSARY WORK.

The other patients wanted cough mixture and dressings of various sorts which I was able to supply from the very scanty materials that were to hand. In less than a week, however, I had settled into my dispensary and begun to have quite a large clientèle. The natives here suffer very much from ulcers on their legs and feet, caused by a little insect called "tekenya," which burrows into the skin, and if not removed it soon sets up inflammation and ulceration. Conjunctivitis is also very common among them—infants' eyes are nearly always neglected until they get quite alarmingly bad. On an average I get between 30 and 40 outpatients daily, and am very often called to see sick people in the village.

### AN UNEXPECTED RECOVERY.

On one occasion one of our workmen asked leave to go and weep for his sister, who was about to die. I suggested that some medicine might do her good, and though he did not think that possible, he said that if I would go with him to see her he should be glad. So I went, and found the patient lying outside her hut—her head supported on her husband's arms, and all her family squatting round watching her. Quite near the neighbours were cooking the rice and goat for the funeral feast, and from the expression on the faces of the prospective mourners I fancied that they were wishing the old lady would make up her mind to depart and let them satisfy their appetites. They made way for me at once, and I knelt by the sick woman and examined her. So far as I could tell she was suffering from acute bronchitis, and though her breathing was fearfully laboured, I thought she would pull through. When I explained my views to the family they did not hide their disappointment, but when they found that I was right and that the old lady did recover they came in a body to thank me.

### DOING DRESSINGS ON DOORSTEPS.

I wonder if any of the readers of THE HOSPITAL are in the habit of doing their dressings (surgical) on doorsteps. One woman I treated went to sleep near a fire and burnt her knee very badly. She was unable to come to me, so I carried the dressings down to her. I found her lying under the broad eaves of her hut in great pain. The wound, which extended from behind the ankle to well up the thigh, going right round the knee, had been covered up with cow-dirt and leaves, and was horribly foul. I sat down on the doorstep, spread a large newspaper as a mackintosh, and commenced operations. A large crowd assembled in the road to watch. This happened every day for six weeks, when the leg was entirely healed.

### A DOCTOR WANTED.

The African cannot as a rule be described as a grateful patient, but occasionally an exception occurs. I have had various presents from people I have treated, always long after they had ceased to come for medicine, such as eggs, a fish (smoked and somewhat high), a comb made of rushes, a turtle's egg, &c., &c. My great need, of course, is a doctor to explain the very many things I cannot diagnose. It is not the most delightful moment I have known when a man breaks his arm, and I have to set it with an improvised splint and strapping that won't strap, and am not quite sure whether it is the radius or the ulnar, or both, that is injured. However, it is a life full of incident. I have said nothing of the Europeans I have to nurse, whose lives under Providence depend occasionally upon the person looking after them; and if anyone in England finds nursing dull or monotonous I recommend her to come out here to Lake Nyasa and spend a year or two.

### To Nurses.

**WE invite contributions from any of our readers, and shall be glad to pay for "Notes on News from the Nursing World," or for articles describing nursing experiences, or dealing with any nursing question from an original point of view. The minimum payment for contributions is 5s., but we welcome interesting contributions of a column, or a page, in length. It may be added that notices of entertainments, presentations, and deaths are not paid for, but, of course, we are always glad to receive them. All rejected manuscripts are returned in due course, and all payments for manuscripts used are made as early as possible at the beginning of each quarter.**

### No. 3 Model School Hospital, Pretoria.

By an Army Sister.

Will you try to picture a lovely old-fashioned garden, covering about 2½ acres, where pepper trees with their feathery leaves and sprays of pink berries, oaks with huge acorns, firs, bamboos, eucalyptus, beeches, Morton Bay figs, magnolias, loquats, and cypress, with all their varied foliage, vie with each other in luxuriance, and combine in themselves the vegetation of temperate and tropical climes. And then think of a wealth of flowers, roses and geraniums, the latter growing into bushes, oleanders white and pink, groves of jessamine, arbours covered with honeysuckle or the vivid magenta blossoms of the bougainvillia; the glorious scarlet flowers of the hibiscus trees, yellow arbutulon, English verbenas and white and lilac petunias, Iceland poppies, daisies, and caleopsis, and many other familiar flowering shrubs and plants which must perforce be nameless. In the centre of the garden stands a fountain whose stone border, prettily carved, is made to hold earth for small plants of bright colour, while round the lowest basin of the fountain, quaintly perched on rough rock-like stones, are tropical flowers. A large plat of grass surrounds the fountain, where any morning when it plays may be seen many different kinds of birds of all varieties of plumage taking a morning bath or searching for food among the flower beds. The paths of the garden, mostly wide and gravelled, where the earth is not its natural red, are bordered by violets, which in South Africa grow to an immense size and a wonderful sweetness. Consider all these bathed in sunshine, with a lovely blue sky overhead and an atmosphere so clear that all objects have a definiteness you seldom see in England, and you have a meagre idea of the surroundings of No. 3 Model School Hospital, Pretoria, where it is my good fortune at present to be working. The hospital, which was at first a branch of the Yeomanry Field Hospital, and opened in the third week of July, was taken over from the Dutch, who had diverted the buildings to their present purpose. As the name suggests, they were originally a school for girls—a large one it must have been, as the hospital accommodates 120 beds, and consists of four separate blocks of different size scattered irregularly in the grounds.

### The Interior.

Almost the largest of these—a bungalow—facing the fountain and the road is used for officers' wards and mess-room, the long verandah, or stoep, running from end to end, making a very nice sitting-room for invalids, and also being very convenient for the sick, who can be taken in their beds, or long chairs, under the shade of the trees on the grass. The same block contains a splendid room for the medical and nursing staff, who mess together, and a few bedrooms for doctors and nurses. The next building was originally a gymnasium, and now makes a fine ward of twenty beds—except for the suspended mosquito-nets and the large surface of glass at one side like a studio, resembling many a ward in an English hospital. The other beds are in a third block, which is partly two-storeyed, and here the wards are numerous and small with an average of three beds each. The more serious cases are, where possible, received in the larger wards for reasons of ventilation.

### The Nurses' Quarters.

Most of the nursing staff sleep in a narrow block of buildings at a small distance from the main buildings. We are particularly lucky in having rooms to ourselves, comfortable beds, and some of the etceteras of civilised life. There is a delightful sense of freedom in walking straight out of the bedroom into the garden; and this makes No. 3 Model School not only one of the most picturesque, but also one of the most comfortable of hospitals. The nursing staff consists of eight, including the superintending sister, Miss Becker, from the London Hospital. The duty hours are from 8.30 to 8 p.m., dinner being at 7 p.m., with three hours' recreation from 2 to 5 in the afternoon. During that time an orderly sister is in charge of all patients—it is a duty undertaken as a rule in rotation by all the sisters—but if there be a supernumerary the others are relieved from their turn, and she takes duty every afternoon, having the rest of the day to herself.

### "A Most Delightful Duty."

There is one night sister—who is on for a fortnight or sometimes a longer period at a stretch. It is a most delightful duty, for the necessary walking in the garden to get from ward to ward in the glorious semi-tropical starlight night is nothing but pleasure. The stillness—there is no traffic after 10 p.m.—broken only by the sounds of night insects, frogs, and the occasional barking of a dog or the galloping of a horse, is a joy in itself, and the beauty of the garden in the moonlight at dead of night is something to be felt rather than explained in words. Of course, there are nights when nothing but a rain-coat, goloshes, and umbrella are possible, and the charms of the garden and night are mitigated by an abundance of sticky mud and pools of water into which one steps unseeing and regretful.

Nominally, a round of the hospital is made every two hours, but the spirit overrules the letter of the law in No. 3 Model School, and often the visits are hourly or more frequent. The work varies from time to time, as in all institutions, being heavy and light by turns. The cases are mostly medical, and, until lately, chiefly enteric.

### Mr. Winston Churchill's Prison.

The two other hospitals under the group named "Model Schools" are Nos. 1 and 2, the former being the building where Mr. Winston Churchill and his fellow prisoners were confined, and from which the former effected his escape. It also contains the wonderful map about 9 feet by 12 feet, drawn on the wall of one of the wards by the imprisoned officers, illustrating the war up to the date of the last comer's arrival. Most of the surgical cases are now sent to No. 1, and this week they have received many from the serious battle near Rietfontein, when the "Fighting Fifth" lost so heavily. No. 2 School is a two-storeyed building and receives the enterics. All three institutions are within three minutes' walking distance of each other, and are now under one principal medical officer, though in every other respect entirely distinct in management.

### No Red Tape.

Before joining the staff of this hospital I had heard rumours of the excellence of the nursing at No. 3. Acquaintance induces the wish that Mr. Burdett-Coutts, who saw the seamy side, could visit this very happy example of a military hospital, in which there is almost a complete absence of red tape. The nursing is entirely by civilian sisters of the A.N.S.R., with the exception of one New Zealander. Work and the nursing of the sick are not overridden by military routine, and cleanliness and order, peace and content, are marked characteristics. I cannot forbear telling how I asked for clinical thermometers, and how, within thirty-six hours—I believe it was really next day—six were obtained and three given to me for my ward, without any signing of blue papers or requisition forms. The same spirit runs through all the nursing details. The hospital is served by R.A.M.C. orderlies chiefly and a few regimental men. Their hours, like those of the Irish hospital, are arranged on civil lines.

### Our Amusements.

The hospital is lighted throughout with electricity, and the garden also. We have a piano in the mess-room, and occasionally in the evening music and singing, which passes time pleasantly for all concerned. One of the features of the day is afternoon tea in the garden. Visitors frequently drop in, and old officer patients come to show sunburn and health after sickness in the wards. Sometimes we have a military band to while away the time with delightful music for two hours in the afternoon: it is so much appreciated by the patients, and No. 3 is then "at home" to its numerous friends.

### The Alexandra Nurses.

(By Our Commissioner.)

### A CHAT WITH COLONEL GILDEA, C.B.

When I called on the Chairman and Treasurer of the Soldiers and Sailors' Families' Association at 23 Queen Anne's Gate I found him busy opening and acknowledging responses to the Princess of Wales's Appeal on behalf of the relatives of the soldiers who are engaged in the war. But Colonel Gildea agreed that the present was an appropriate time to refer to the work carried on by the nursing branch of the Association. Fitly enough, the nurses who work under it are called after the President, and bear the name of Her Royal Highness. Moreover, as Colonel Gildea says, no nurse is entitled to the honour of being called an "Alexandra" nurse who is not solely employed by the Association, and does not wear the prescribed uniform.

### The Training.

"And about the training?" I asked.

"Our system is the same as that of the Queen's Jubilee Institute; and in addition to the three years' training in hospital and six months' experience in district nursing which we require, we like all our nurses to possess the L.O.S. certificate. One of their duties often is, with the sanction of the Committee or medical attendant, to attend cases after childbirth where skilful nursing is considered necessary. No Alexandra nurse is allowed to act as midwife.

"What are the duties generally?"

"The daily visitation of the wives and families of soldiers and sailors. As to the controlling authority, the nurse is under the direction and control of the President and Committee of our local division."

"Do they arrange the hours of work?"

"A nurse is not expected to work for more than eight hours daily, but the Committee decide the times. The eight hours are not extended save under exceptional circumstances."

### The Uniform.

"Of course a uniform is worn."

"Always when a nurse is on duty. The dress is plain cotton Oxford grey washing material, with white linen collars and cuffs and two square pockets in front. A white square apron is worn over it with bib and straps, but no pockets. The waist belt is navy blue in the centre and army red on either side, and is fastened by a gilt regulation buckle in front."

"And the bonnet?"

"Blue fancy straw, 'Marie Stuart,' trimmed with wide regulation ribbon similar to the waist belt. White muslin strings are worn, but no veil. With permission of the local committee a straw hat with regulation ribbon may be worn in summer. The summer dress is of dark blue Foulé, fastened down the front with the regulation buttons, and it is compulsory that the bottom of the skirt should be at least six inches from the ground. The circular cape is of the same material, with turn-over collar lined with red silk and fastened down the front with regulation buttons. In winter a cloak of dark blue Melton cloth is prescribed."

"What is the cost of the uniform?"

"All that is essential—£6 10s. Directions for self-measurement are supplied by Messrs, Debenham & Freebody, from whom alone the uniform can be obtained."

### The Work.

"I presume that the work of the nurses does not clash with that of the nurses in hospital."

"No nurse is permitted to attend any form of sickness that should more properly be treated within the wards of a hospital. A register of cases is kept by each nurse, which is always open to the inspection of any member of the local committee, and one of the regulations is that a nurse shall only attend cases authorised by the Committee or to whomsoever they may depute their authority."

"But if she is summoned to a case of emergency?"

"Then she is required immediately after her first visit to report the case to the Committee or their representative."

"Is there any night duty?"

"Only under very special circumstances, and when proper provision can be made for the efficient nursing of other cases. The nurses are responsible for the personal cleanliness of each patient under their charge, and they are enjoined to improve the general surroundings of the patient."

### Free Services.

"May a nurse give relief?"

"In cases of extreme urgency she may administer nourishment only, and she is expected to report such cases at once to the Committee."

"Are her services afforded to the patients quite gratuitously?"

"Yes, in all instances; and she is not allowed to accept presents of any kind from patients or their friends, whether during illness, or after recovery, or death. No patient is under an obligation to make a contribution for benefits received, but should any one desire to do so, it should be sent to a member of the Committee for the work of the Nursing Branch of the Association."

"Supposing that the eight nurses are not occupied in nursing the sick?"

"When a nurse is not engaged in tending the sick, she visits the wives and the families of soldiers and sailors in her district in turn, and gives general hints and advice on matters of health and hygiene."

### Holidays and Badges.

"To what holidays is a nurse entitled?"

"Three weeks in the year. She should also, if possible, have half a day off each week, or a day every other week."

"What are the regulations as to badges?"

"A bronze badge is worn round the neck by every nurse for three years, when a silver badge is given in exchange. The bronze badge is returnable to the Association, but the silver one becomes the property of the nurse on the completion of five years' continuous service. Otherwise, it is returned. If a nurse loses her badge, she has to pay for a new one."

### The Branches.

"When did you start your nursing branch?"

"In 1892. The first nurse appointed was sent to the Curragh. Since then we have established branches in Dover, Aldershot, York, Malta, Devonport, Stonehouse, Cairo, Canada, the Mauritius, Natal, and Colchester."

"Can you give me any particulars of the work done at the stations?"

"In the Curragh our nurses had in the year, according to our last returns, 109 cases in 1899 and, paid upwards of 1,400 visits. At Dover the cases were 143 and the visits 1,145. We have three nurses at Aldershot where our president is Lady Audrey Buller. The cases in the year were 615 and the number of visits paid was 9,539. At York, where we work in connection with the York Home for Nurses, there were 89 cases and 1,171 visits. We have three nurses at Malta, and casual visits are found to be invaluable there. At Devonport the nurse had 190 cases and paid 2,849 visits, and at Stonehouse the cases were 126 and the visits 2,285. Our Cairo nurse had no less than 514 cases and paid nearly 10,000 visits."

CANADA AND NATAL.

"In Canada the nurses of the Victoria Order of Nurses work for us on condition of a half-yearly payment of £17 10s. guaranteed by us."

"Does the arrangement answer?"

"Admirably. Lady William Seymour, our president, reports that the nurses are most kind and attentive to their patients. In the Mauritius our nurse's duties are confined to the hospital, where she has served as matron. At Pietermaritzburg, our Natal branch, there were 572 cases, and the nurse paid 4,509 visits."

"I may mention," continued Colonel Gildea, "that after the wives and families of the soldiers at Maritzburg had been sent home, our nurse has been made use of by the authorities for nursing the sick and wounded soldiers, especially in the early stages of the war, before the relief of Ladysmith. Her services were much appreciated. Our last branch was started at Colchester in 1899, and in six months 723 visits were paid. The services of our nurse there have lately been more utilised for cases 'off the strength,' as well as for those 'on the strength.' The salaries vary according to circumstances."

A NURSING HOME AT THE CURRAGH.

"Did you make any additional appointments in 1900?"

"No, we have been very quiet in the nursing branch lately, owing to the pressure upon the Association in other directions; but the first nurses' home is now being completed at the Curragh Camp. It is called, by special request, the 'Alexandra,' after the Princess of Wales, who takes the very greatest interest in the branch."

"What is the cost of it to be?"

"Upwards of £900. The amount represents the proceeds of the fête held at Chelsea Hospital in 1897. The Home, which is placed in the very centre of the camp, on ground given by the Government, has a sitting-room, a waiting-room, the usual offices, and three bedrooms. We hope that it will be opened during the present month."

"How much does the nursing branch cost the Association?"

"A little over a thousand pounds a year. We shall be only too glad to extend its operations as our means permit. But we depend, as you know, entirely upon the support of the public."

### The Nurses' Bookshelf.

NURSING AND HYGIENE. By R. LAWTON ROBERTS, M.D. Third edition. (H. K. Lewis, 136 Gower Street, W.C. Price 2s. 6d.) Crown 8vo. Pp. 223. 71 illustrations.

THIS volume is a third edition of a series of eight lectures given ten years ago. There are two difficulties attaching to the publication of lectures. A good lecture style is often not good literary style, and there is the difficulty of bringing the lectures up to date. If Dr. Roberts' book had been dealing with the technique of the art (as nursing is understood in the hospitals), a period of ten years would have rendered the work obsolete. It is, however, addressed to novices, and is intended more particularly to supplement instruction in ambulance work—a very different thing from routine hospital work—and consequently the lapse of ten years makes little or no difference. The chapters dealing with ablutions, ventilation, cleanliness, food are very good as an initiation into nursing. The ignorance on these subjects is so prevalent that the "glimpses into the obvious" will be anything but a work of supererogation. The only attempt, and that not wholly successful, to bring the book up to date is in the appendix, for we find no mention of serum-therapeutics, of the mode of injection of the latter, of the administration of oxygen in lung and other disorders, or of the immense strides made in aseptic as well as antiseptic surgery and treatment of wounds. Some remarks on the recent epidemic of enteric among the troops in South Africa in connection with the theory and practice of inoculation against the disease, together with an account of the Mauser, Lee-Metford, and expanding bullet wounds, would have been of absorbing interest to the ambulance section of the reading public, to whom this book is professedly addressed, and at the same time would have brought it into line with the latest observations and theories in war medicine and surgery. The exhaustive trials in the *Lancet* with reference to filters have evidently been followed by the author, who puts in the conclusions of the *Lancet*, i.e., that the Pasteur-Chamberland and the Berkefeld are the only two filters which will successfully arrest pathogenic bacilli. This gives point to the remark we made at the commencement—viz., that ten years is a very long period in the accumulation of knowledge on any one subject; and in this connection we would suggest in a further edition fuller directions on the dressing of wounds and preparation for operations according to strictly aseptic methods, a detailed direction on the use of the catheter (an important nursing detail which appears to be omitted altogether in this work), artificial feeding by tube and large nutrient enemata, together with the names and descriptions of the various lotions and antiseptics brought up to date—e.g., lysol, biniodide of mercury and electrozone—none of which we can find mentioned in this work. In conclusion, we take exception to the remark in the preface to the third edition that "instruction in nursing is now provided for men in the same way as for women." Can the St. John's ambulance orderly, however good, be equal to the fully-trained sister or nurse from a big general hospital? The answer could be given by our sick troops in South Africa.

THE MYSTERY OF LADYPLACE. By CHRISTIAN LYS, author of "The Fortress of Yadasara," "The Hepsworth Millions," &c. (London: Frederick Warne & Co.) Price 6s. net.

FOR the fairly strong-minded this is the sort of volume with which to while away a long railway journey, or to occupy a lonely evening by the side of the fire. From first to last the interest is admirably maintained, and the author skilfully reserves until the last the solution of a problem which increases in perplexity as the story proceeds. We certainly do not propose to spoil it for those whose affections centre on sensational fiction of a quite unobjectionable character. They will prefer to find out for themselves by whom the diamonds belonging to Mr. Wynton's ward were stolen, and other secrets which Mr. Christian Lys provides for the delectation of his readers. There are but two points of criticism which suggest themselves to us. One is that the Jew, Dr. Schreiner, is allowed to talk too much broken English. The other is that an author who can give us such a delightful bit of romance as the love-making between Robert Wynton and the girl he has loved from childhood, is capable of a better class of work than "The Mystery of Ladyplace." He has shown this before, we believe, in his other novels; and though it is unquestionable that he hits the modern taste in his new book, without pandering to any unhealthy cravings, we gather from the pages before us that he could heighten the charm of a clever plot more completely, than he has done on the present occasion.

CASSELL'S MAGAZINE for January. Published by Cassell and Co.

THE feature of *Cassell's Magazine*, which commences a new volume this month, is Mr. Rudyard Kipling's striking new story "Kim." It is a story of India, where the author was born, and where he started his reputation. Some capital original illustrations are supplied by his father, Mr. Lockwood Kipling, and Mr. E. L. Weeks.

### Where to Go.

GLOBE THEATRE, NEWCASTLE STREET, STRAND, Thursday, January 24th, Friday, January 25th, at 2.30.—Performance by amateurs of "The Home Secretary," by R. C. Garton, in aid of the Grosvenor Hospital for Women and Children.

### Preparations of Beef-tea for the Sick.

By HELEN TODD, Matron, The National Sanatorium, Bournemouth.

(*Concluded from page* 171.)

Patients who, owing to the nature of their illness, can only be permitted fluid forms of nourishment frequently become very weary of the oft-repeated milk and eggs, and hail the variation of beef-tea with relief. It is therefore still one of the most popular items in a dietary restricted to liquids, although, as we have seen, the home-made article contains very little nourishment, even when well made, and is absolutely useless when made by a careless cook.

### An Alternative to "Bought" Beef-tea.

In order to meet the prejudices of those who still object to "bought" or machine-made beef-tea the following recipe has been carefully compiled. It will be found free from the many objections in those recipes alluded to in last week's paper, and far more satisfactory in every respect. To make 1 pint beef-tea allow 1¼lb. of topside of beef, as freshly killed as possible, and free from all bone, fat, skin, and sinew. Chop it up finely, being careful to cut across the "grain" of the meat. Put it into a covered earthenware jar containing one pint cold water which has previously been boiled; add ʒss salt. Let the whole stand in a cool place for 1½ hour before cooking. If the oven can be regulated so that its heat can be kept at 158° Fahr. the jar may be placed in it and left for three hours; but if it has to be constantly opened, or its heat varied for the requirements of the family dinner, the jar containing the meat and water must be put instead into a large pan of water the heat of which must be regulated so that the contents of the jar be kept as nearly as possible at 158° Fahr. for three hours. Care should be taken that the water in the pan is always at a sufficient height to surround the contents of the jar; and when necessary to add more the water must first be brought to the required heat. After the cooking process is completed the jar should be at once removed to a cool place, and there left undisturbed until its contents are quite cold. The particles of fat, having now become congealed, can be easily removed in a solid mass and the beef-tea strained through a fairly coarse wire sieve, the finer particles of the meat being rubbed through by means of a wooden spoon.

### The Addition of Vegetables.

Many recipes advocate the addition of vegetables—such as turnips, carrots, onions, and celery—in order to improve the flavour of the beef-tea. This at once alters its character from that of a beef-tea proper to that of a soup, and must never be allowed in cases of severe illness without the doctor's permission. The nurse must especially be on her guard against these unauthorised additions in cases where the patient is suffering from diabetes or any illness with his complication, carrots, parsnips, and onions being the very vegetables which are strictly prohibited where this is the case on account of sugar-forming properties. If a change of flavouring be imperative celery can be used, it being one of the few vegetables which may be eaten by diabetics.

### When Beef-tea Should Not be Given.

All preparations of meat and its extracts are forbidden in cases of acute Bright's disease. Patients suffering from nephritis at once show an aggravation of symptoms after taking any beef-tea or meat extract; this, consisting, as we have seen, of as much of the albuminoids as can be extracted from the muscle, is rapidly absorbed by means of the blood capillaries of the stomach into the circulatory system, and its elimination by the kidneys seriously increases the strain upon them. Such articles of diet, therefore, "should be strictly excluded from the dietary of the albuminuric."[1] In the febrile stage of acute rheumatism beef-tea must be avoided, and only weak solutions given as the patient progresses in convalescence. The nurse should never on her own responsibility give beef-tea to patients with any gastric ulceration, nor to children suffering from any form of meningitis.

### A Recipe for Acute Diarrhœa.

In cases of acute diarrhœa it is usual to thicken the beef-tea with arrowroot, sago, or tapioca; for such patients the nurse will find the following recipes invaluable:—

*Beef-tea with Arrowroot.*—Arrowroot ʒss, beef tea ℥x. Mix the arrowroot to a smooth paste with a little cold water. Have the beef-tea boiling in a saucepan, and pour it quickly upon the paste; stir thoroughly and serve. (If the beef-tea be poured on slowly the mixture will become lumpy, and no amount of stirring will ever make it smooth.) If no beef-tea is to be had, and the mixture is wanted urgently, bovril may be used instead; it should be mixed with the paste of arrow root and water, and ℥x boiling water poured on in the same manner as the beef-tea in the above instructions.

*Beef-tea with Sago or Tapioca.*—1. Proceed to make the beef-tea as directed at the beginning of this article, adding tapioca (in the proportion of ʒij to ℥xx of beef-tea) to the chopped meat and water one hour before cooking. 2. If the beef-tea be already made, add the tapioca in the above proportion and let it simmer gently for three hours, giving an occasional stir to prevent the grain sticking to the bottom of the pan or burning.

N.B.—All grain, whether tapioca or sago, must be washed in cold water before being used.

### Beef-tea Custard.

In cases of cancer of the rectum Sir Lauder Brunton advises for some patients beef-tea custard. The recipe he recommends consists of three eggs to beef-tea ℥xx. The whites of two of the eggs and the yolks of three are to be well beaten, first separately and then together, mixed with the beef-tea, and then placed in glasses, and the resulting custard set by standing in hot water.[2] In cases of exhaustion, shock, and collapse beef-tea is an excellent vehicle for the administration of brandy, the taste of which also it disguises to a certain extent.

### Beef Essence.

In severe cases of this description, when it is difficult to persuade the patient to take any form of nourishment, the nurse will find "beef essence" useful as a more concentrated food. This is made by taking 2 lb. of lean beef from the best part of the topside, cutting it up very finely and putting it into an earthenware jar with a little salt and ℥ij cold water. A bladder should be tied tightly over the jar, and the whole stood in a pan of boiling water, and kept at an even heat of 158° Fahr. for six hours. The liquor should then be poured off, allowed to cool, and the fat skimmed off. Beef essence made as above should jelly when cold: many patients prefer it iced, and can enjoy it when ordinary beef-tea would make them sick.

### An English Form of Food.

It is an interesting fact that beef-tea, as we know it, is a purely English form of food for the sick. On the Continent bouillon or consomme takes its place both in the hospital and private house. I have been in correspondence with several ladies, both French and German, on the subject: their recipes differ totally from ours in that they invariably add large quantities of vegetables, in many cases partly stewed in butter before being added to the broth, so that the resulting food is far more of the nature of a stew or thick soup than what we consider a beef-tea.

[1] Burney, Geo., Man. Med. Treat., p. 271. [2] Action of Medicine, p. 446.

### Echoes from the Outside World.

### AN OPEN LETTER TO A HOSPITAL NURSE.

On December 22nd there appeared in the *Hospital Nursing Mirror* a report of a speech on "District Nursing" in which Mrs. Creighton said that when there was sickness in the family one realised that the comforts of life, and everything that money could give, were often unavailing, and that at such times the sense of the pain of the world was almost unendurable. When she spoke on that occasion her hopes of her husband's recovery were bright. But, unhappily, they faded away entirely a few days ago, and on Monday afternoon the Church of England mourned the death of a prelate who combined goodness with greatness, and Mrs. Creighton and her children the loss of a husband and a father whose public virtues had more than their counterpart in the family circle. Splendid as the gifts of the deceased prelate were, and marvellous as his versatility was, nothing, neither his learning, his intelligence, his eloquence, nor his humour, endeared him to all sorts and conditions of people so much as his unfailing kindness of heart, his readiness to sympathise, his genius in putting himself in the position of those who sought his counsel and assistance. In that darkened home at Fulham there must be the consolation that the life sacrificed on the altar of duty is with universal consent acclaimed as one of the noblest, richest, and most useful that has been spent in the service of others.

It is seldom there is such a sensational wreck as that of the *Russie* last week. To be only 400 yards from the shore, to see hundreds of people stretching out their hands to help, amongst whom were some of the dearest relatives of the passengers, to hear their voices, and yet to feel that death might come at any moment, must have been torture indeed. About half-past four in the morning of Monday the ship struck on the beach at Faraman, near Marseilles. Any attempt to launch boats would have been madness, so the captain persuaded his passengers to go below and await assistance from the shore. But that assistance was long in coming. The rocket apparatus failed every time; the ropes and cables which were sometimes successfully started between the would-be rescuers and the would-be rescued snapped again and again; little by little the sea gained the supremacy and took possession of one room after another; day after day food got scarcer, and the water supply ceased; and yet the waves rushed on, and every boat which tried to reach the wreck, except one which poured some oil on the troubled waters, was obliged to turn back. Then, when hope was almost dead, the angry sea ceased to rage, the wind dropped, and a life-boat appeared at three o'clock on Friday morning, and the first boatload of women and children, including a baby six months old, were taken ashore. Madame Fredo, the stewardess, showed the sterling stuff of which some women are made; she refused to leave with the ladies, maintaining that she was one of the crew, and as such should leave last. Another instance of unselfish heroism was that of a Bavarian soldier belonging to the Foreign Legion. There was an enormous leak in the side of the vessel. This was stuffed up with mattresses and cabin doors, and so that there should be no chance of disaster owing to the impromptu repair giving way, he remained at his post for more than thirty hours, standing in the water all the time dressed only in shirt and drawers. In reading the accounts I could not help wondering whether there were many total abstainers on board, and if so, how they behaved. The second day of the accident the water-supply was exhausted, and with the exception of some aërated waters, the passengers and crew had all alike to drink wine, of which there appears to have been plenty. Champagne and even heavier alcoholic drinks were not the best form of beverage for men and women worn out by want of proper food, want of sleep, and anxiety, and it is not to be wondered at that some of the shipwrecked people are said to have drowned their sorrows in jollity or insensibility. But even the most rabid teetotaller would probably, after some hours' experience, prefer wine to increasing thirst, and, in moderation, it must have been a good temporary tonic. The ménu of the last meal taken on board must have made the victims of the disaster especially grateful that they did not have to repeat the fare for breakfast next morning. It was unlimited wine, raw artichokes, and tangerines.

Those who believe that first breaches of the criminal law, unless they are of the most deplorable character, should be treated lightly, and with the idea of giving those who commit them a second chance, will find their faith strongly justified by the figures contained in a return just issued by the Home Office. From this return it appears that of 2,191 persons released on their own recognisance under the First Offenders Act in three years, only 257 are known to have been subsequently convicted of a fresh offence and to have been called up to receive judgment. These figures represent the cases at fourteen London public courts, and the only unsatisfactory feature about them is that at some of the courts, such as Woolwich, Clerkenwell, and Marylebone, the quality of mercy has not been exercised as it has at Marlborough Street, Worship Street, West London, and Southwark. It may be hoped that with this evidence of the value of the Act before them, magistrates, whether in town or country, will on every possible occasion be careful to enforce the admirable policy of one of the most enlightened measures which has ever been added to the Statute Book.

I do not know if you agree with me that it is a desperate pity to take mere babies to a pantomime which is too long for their powers of endurance, and too up-to-date for their powers of comprehension? It tends so much to spoil their enjoyment later on, and of all the dreadful creations of the present age, I think that the "blasé baby" is the most to be deplored. To my mind a circus is preferable to a pantomime as a treat till the little folks are seven or eight years of age. And accordingly, as we had two little nieces to stay for a day or two, we went to the Crystal Palace to see the Imperial Russian Circus now performing there. It is a very good entertainment, with a great deal that is clever, and a fair amount of originality. The juggler on a bare-backed horse deservedly won special applause, and the sentiment expressed by a little boy next me I entirely echoed, "I do like to see him throw up the things and catch them in that hole in his head." It sounded as if the poor man wanted assistance from a surgeon and a nurse, but the hole in his head was in reality a little receiver, strapped on so that he might carry out the novel cup and ball trick. Also the three Wortleys in the flying trapeze act were capital, and the children liked to see the acrobatic bears go through their performance. Occasionally there was a growl or two which suggested being "naughty," and of course pleased the youngsters, though I believe, as a matter of fact, trained animals are taught to growl at certain intervals as they are taught to dance. It enhances the value of the show as far as the public are concerned, because it suggests danger. But this element is reduced in reality to a minimum at Sydenham.

As perhaps you know, there is in connection with the National Society for the Prevention of Cruelty to Children a League of Pity, whose object is to promote amongst happy children the service of the unhappy. On Tuesday afternoon a treat was given at the King's Hall of the Holborn Restaurant by its members to a number of children in cases under the supervision of the Society, whose lives have been thereby rendered secure with their parents in their own homes, upwards of 600 guests being present on the occasion. Their enjoyment, as well as that of their young hosts, who with their friends watched the proceedings from the galleries, was very obvious. Indeed, the delight of youngsters whose lives are so seldom brightened by any pleasure must have more than compensated the givers of the treat for their thoughtfulness and liberality.

### Appointments.

MELBOURNE HOSPITAL, VICTORIA, AUSTRALIA.—Miss Annie Burleigh has been appointed matron. She was trained at St. Bartholomew's Hospital, London, and has since been matron of the City of London Hospital for Diseases of the Chest, Victoria Park, E.

BRIDGE SCHOOLS, WITHAM.—Miss Emily Baker has been appointed matron. She was trained at the Hospital for Sick Children, Great Ormond Street, and has since been resident nurse at Dean Close Memorial School, Cheltenham, and the City of London Freemen's Orphan Schools.

ISOLATION HOSPITAL, WALTHAMSTOW. — Miss Louisa Pratchett has been appointed matron. She was trained at the Victoria Hospital, Folkestone. Subsequently she was staff nurse, and has since been staff nurse at the Borough Hospital, Folkestone, and for four years charge nurse at the Isolation Hospital, Willesden.

MEMORIAL HOSPITAL, JARROW-ON-TYNE.—Miss Margaret Storey has been appointed matron. She was trained at the Leeds General Infirmary, where she was afterwards operation theatre sister and ward sister. She left Leeds in order to become matron of the Fleetwood Cottage Hospital, where she remained for nearly two years.

CHALMERS HOSPITAL, EDINBURGH.—Miss F. Strange has been appointed matron. She was trained at Salisbury Infirmary, and has since been staff nurse at the Royal Hants Hospital, Winchester, assistant nurse at Tiverton Infirmary, charge nurse at Salisbury Infirmary, night superintendent and sister-in-charge of Chalmers Hospital, Edinburgh.

LAMBETH WORKHOUSE INFIRMARY.—Miss Rosalie Mansell has been appointed superintendent nurse. She was trained at the Homœopathic Hospital, Great Ormond Street, and the General Lying-in Hospital, York Road. After her probationership she was staff nurse and charge nurse at the Homœopathic Hospital, charge nurse at the Eastern Fever Hospital, Homerton, did surgical work at the Hanover Hospital, George Street, Hanover Square, and had charge of the Smallpox Hospital, Southend-on-Sea. She has also done six years' private nursing.

### Minor Appointments.

HENLEY-ON-THAMES.—Miss Cordelia Duffield has been appointed parish nurse. She was trained at St. George's Hospital, where she was subsequently staff nurse and sister.

ISLINGTON INFIRMARY.—Miss Harriet Richards has been appointed assistant nurse. She was trained by the Meath Workhouse Nursing Association at the Home for Invalids, Aubert Park, Highbury.

WOLVERHAMPTON GENERAL HOSPITAL. — Miss M. F. Urquhart has been appointed sister. She was trained at Charing Cross Hospital, and has since been charge nurse at Victoria Convalescent Home, Broadstairs, and sister at East Dulwich Infirmary.

DISTRICT NURSES' HOME, BARRY, GLAM.—Miss Laura Tyler has been appointed district nursing sister. She was trained at the Bristol Royal Infirmary, and remained there five years. She has since been charge nurse at Weston-super-Mare and the City of London Chest Hospital, matron of the Chalfont St. Peter Cottage Hospital, assistant matron Stratford-on-Avon Fever Hospital, &c. For the last three and a half years she has been engaged in district nursing at Levenshulme.

### Presentations.

ST. SAVIOUR'S INFIRMARY, EAST DULWICH.—Miss Bourdillon, on resigning her post as night superintendent, was presented by the staff with a handsome case of silver-backed brushes, showing the high esteem in which she was held by all who knew and worked with her.

ISOLATION HOSPITAL, WILLESDEN.—Miss Pratchett has been presented with a handsome vase, a set of afternoon tea-spoons, and a five o'clock tea service from the doctor, matron, and nursing staff on her retirement in consequence of her appointment to the post of matron at the New Isolation Hospital, Walthamstow. Miss Pratchett, who has been at Willesden four years, leaves very many friends among her colleagues, and at an afternoon tea given in her honour she received the good wishes of all. Later in the evening the nurses had a little dance.

MILL LANE HOSPITAL, LISCARD.—On New Year's Day Miss Malcolm, matron of Mill, Liscard, was the recipient of several tokens of appreciation and esteem from the nurses and servants on the staff. The charge nurses gave her platinotype views of the Lake District in oak frames; the assistant-nurses and probationers, silver sugar sifter, fruit stands, afternoon tea-pot, and letter rack; and the maids a silver toast rack. The Christmas festivities were much appreciated and enjoyed by all. They included a dance given to the nurses by the matron and committee, to which the attendant medical staff and a number of private friends were invited.

### Two Hospital Entertainments.

THE authorities of the West End Hospital for Paralysis, &c., provided an excellent entertainment for the patients on Saturday afternoon, when, after tea in the wards, they were taken down to the out-patients' department to witness a performance of conjuring and ventriloquism by Mr. John Warren. In their scarlet frocks and white pinafores the little ones made a pretty scene, surrounded by the smiling faces of nurses and visitors, who all combined to make the entertainment a success. The one note of discord was the howl raised by the babies when Mr. Warren introduced his exceedingly life-like dummy; and even this may be taken as an indirect compliment. The sounds of grief were, however, quickly hushed, and the performances of the lively "Joey" caused great merriment. Festoons of Chinese lanterns hung from the ceiling of the room, giving a bright and pretty effect.

At the National Orthopædic Hospital, Great Portland Street, the patients were mightily enjoying themselves on Saturday evening, with a large tree, on which were presents for everyone, from the matron down to the hospital cats, for whom someone had provided small mechanical mice. The ward was well filled with children and visitors; and the few adult patients at present in the hospital had hobbled or hopped down to receive their gifts and listen to the singing and speeches. Not that there was much of the latter; it was only for a few moments that the Rev. E. Grose Hodge, the chaplain, asked for silence, and the whistles and trumpets and chatter were hushed while the committee were thanked for providing the entertainment. Mrs. Cooper especially had been very energetic in collecting books as gifts to the patients and staff, which were especially welcome, and on the selection of which much care had been bestowed. Mr. J. R. Cooper responded, and Mrs. Cooper said a few bright and cheerful words to the patients and wished them all a very happy New Year. Mr. Tresidder, the secretary, said that the idea was started some three years ago that the patients and their friends might like to collect funds at Christmas by means of collecting cards; the first year the amount was £9, last year it was £22, and this year, so far as he could say at present, it was not below that figure. The ward was prettily decorated with Chinese lanterns. Every patient was present, and it will be long before Saturday's entertainment is forgotten. "They have been talking about it for weeks," said one of the nurses, "and I expect it will form the topic of conversation for weeks to come."

### Resignation of the Matron of Chelsea Infirmary.

THE news that the Chelsea Infirmary is about to lose the services of Miss de Pledge, who has so ably held the post of matron in that institution for the past twelve years, has been received with great regret by the members of her nursing staff, no less than by the Guardians themselves, and by all those who are at all acquainted with the excellent work she has done in her own department during her term of office. The reputation of the Chelsea Infirmary as a training school for nurses, started on a firm basis by Miss Mollett, has steadily risen under Miss de Pledge's matronship; a very high standard of efficiency has been maintained, and the esteem and affection with which the retiring matron is regarded by her workers affords eloquent testimony to the success of her labours in the cause of trained nursing. Only those who have personal knowledge of the circumstances can realise how strenuous the work has been, and how great, at times, the difficulties which Miss de Pledge has had to fight, although she has had the unwavering support of the majority of the Board of Guardians, who fully appreciated the advantages accruing to the infirmary from her management.

The interesting announcement of Miss de Pledge's approaching marriage to Mr. Latter, Vice-Chairman of the Board of Guardians, was made at the meeting last week, after her letter of resignation had been read, and the following resolution was proposed by the Rev. Lawson Forster, seconded by Mr. Blore, and carried unanimously: "That we accept the resignation of Miss Josephine L. de Pledge, the Matron of the Infirmary, with profound regret, and that in doing so we would take the opportunity of recognising and placing on record our high appreciation of the eminent services rendered by Miss de Pledge to this institution during her twelve years of service. At the same time we would congratulate Miss de Pledge on her contemplated union with one of the most esteemed members of our Board, believing as we do that under God her future life will be attended with much comfort and happiness."

Miss de Pledge's numerous friends will warmly echo these congratulations.

### Everybody's Opinion.

### SO-CALLED TRAINED MALE NURSES.

"M." writes: Will you allow me a small space in your valuable paper to draw the attention of the public to the imposition practised by some nursing institutions which advertise widely trained male nurses. The public should be made acquainted with the fact that these men are merely lunatic attendants, and most of them are absolutely ignorant of the simple rudiments of nursing. They are sent to cases of pneumonia, typhoid, and other serious cases of sickness which require the most gentle handling and perfect knowledge of nursing. The public would do well to consider the responsibility they take upon themselves by engaging incompetent nurses for such serious cases. If before employing a male nurse the friends of the patient would insist upon seeing testimonials from doctors showing efficiency for nursing physical cases, not mental, it would mitigate the danger patients are constantly exposed to by incompetent nursing.

### SWALLOWING A "DUMMY TEAT."

"EPICENE" writes: The following case has just come under my notice, and so I venture to send you particulars of it:—On Sunday, December 23rd, a mother suddenly missed her baby's "dummy teat," and all efforts to find it were unsuccessful. From that time up to Friday, December 28th, the little one was much upset with vomiting and diarrhœa; it vomited food as soon as taken, and appeared much worse on lying down. As the mother had been confined within that time and the child taken charge of by a neighbour, she naturally concluded it had been given Christmas fare and unsuitable food, and hoped to correct the error by giving it a powder. On Friday afternoon, however, the little one had a more severe attack of vomiting than usual, and brought up what appeared to be a lump of fat, but on examination it proved to be the missing "dummy teat" covered with a thick white substance. Immediately the little one recovered his bright spirits and started to play, in marked contrast to the listlessness and fretfulness up to the moment of vomiting the foreign body, which the mother firmly believes must have been swallowed on the day that she first missed it.

### THE RULES IN PRIVATE NURSING.

"CANDID" writes: I have been a private nurse for many years, and have never questioned the propriety of sleeping in a male patient's room when circumstances permitted and necessity demanded my presence there. A conscientious, whole-hearted, and devoted nurse, who thoroughly understands her work, will be guided by commonsense and a sense of duty, and will not consult her own feelings. In the still hours of the night, all the little pains and weaknesses attending illness are felt more acutely than during the day, and sometimes a nurse's presence has a soothing effect on a patient, even when she is not actually required in the sick-room. A careful, anxious nurse will be willing to sacrifice her own comfort in the interest of her patient. We all agree with Jesmond that it is better to consult a doctor's wishes with regard to the hours for medicine. When "A Watcher" has had a little more experience in private nursing she will hardly think it worth while to raise such questions.

"E. J." writes:—I think it is a pity that when a young nurse asks anything about her work in the *Mirror* she is so often told by other nurses that she must have been badly trained to ask such questions. It must be most discouraging for a new beginner. We should remember that we have all been young and inexperienced, and help each other as much as possible. Every nurse knows that private nursing is different to hospital nursing. I quite agree that the doctor ought to be consulted on all matters of feeding and medicines for the patient. But we cannot always detain a busy doctor to consult him about our sleeping accommodation. When a patient is very ill a nurse would naturally sit up with him. It is quite unnecessary for a nurse to sleep in a man's room during convalescence. He is quite capable of ringing a bell if it is put within his reach. I get an electric bell and put the button under the pillow, or within reach, and induce the patient to promise to ring when he awakes so that the nourishment can be given. If I am in a house where it is essential to economise, and there is not an electric bell, I get a hand-bell and tie a piece of string to it, long enough to reach from my room to the patient's, then I tie the other end to the head of the patient's bed; if I can't fix it so that it rings I put it where it will trail on the boards when the string is pulled.

### York County Hospital.

A VERY successful variety entertainment was given on Friday last by the nurses and probationers of York County Hospital. It opened with a Chinese sketch, taken from Bird's Chinese novel "The Seeker," the costumes and scenery showing to great effect in the darkened room. Plantation songs which followed were a great feature, and caused much merriment, some having blacked themselves so well as to be quite unknown to their fellow nurses. Romberg's toy symphony, given by twelve nurses in early Victorian costume, was a great success, being twice encored. Among other items were "The Backward Child," a humorous recitation given in character, wax-works, and some capital songs. After the National Anthem many hearty cheers were given by the patients; and so much pleasure appeared to have been derived that all must feel more than repaid for any trouble incurred.

### For Reading to the Sick.

RESIGNATION.

One adequate support
For the calamities of mortal life
Exists—one only; an assured belief
That the procession of our fate, howe'er
Sad or disturbed, is ordered by a Being
Of infinite benevolence and power;
Whose everlasting purposes embrace
All accidents, converting them to good.
*W. Wordsworth.*

Think not of what is from thee kept;
Think rather what thou hast received;
Thine eyes have smiled if they have wept;
Thy heart has danced if it has grieved.
Rich comforts yet shall be thine own;
Yea, God Himself shall wipe thine eyes;
And still his love alike is shown
In what he gives and what denies.
*H. S. Sutton.*

It is not the sufferings but the cause, which makes the martyr. God delights not in sufferings, but He delights in the brave and patient love with which the martyr suffers. Happy are they who share the honour and glory of the martyrs! But this is refused to no one by Him who commands us to take up our cross daily and to follow Him. For a life of self-denial is a martyrdom and calls for the martyr's fortitude.—*W. B. Ullathorne.*

It would be well if we were in the habit of looking at all we have as God's gift, undeservedly given, and day by day continued to us solely by His mercy. He gave; He may take away. He gave us all we have—life, health, strength, reason, enjoyment, the light of conscience; whatever we have good and holy within us; whatever faith we have; whatever love towards Him; whatever prospect of Heaven. All comes from Him. He gave; He may take away.—*J. H. Newman.*

There is comfort in the certainty that, though the limit of any trial be hidden from me by God, that limit is prefixed, and is all along well known; the end is planned and adjusted from the beginning. As the hairs of our head, so the throbs of our agony are all numbered.—*Christina Rossetti.*

Thoughts of the Christ should rise at every turn,
And hold us all day long:
Alone, or when in crowds, each heart should hear
That blessed undersong,
Which upon Nature's harp is whispering still
Its soft undying strain,
Moving the wakeful soul with deep desire
To see His Face again.
O hear it, ye, on whom His gracious Hand
Has made the sacred sign,
The cross of suffering—who have meekly bowed,
To bear that brand Divine.—*C. M. Noel.*

GOD maketh a great silence that we may hear distinctly the softest whisper of the still small voice, and He maketh a great darkness that we may be able to discern the least and farthest of His stars of truth.—*V. W. Gregory.*

### Notes and Queries.

**The Editor is always willing to answer in this column, without any fee, all reasonable questions, as soon as possible.**
**But the following rules must be carefully observed:—**
1. **Every communication must be accompanied by the name and address of the writer.**
2. **The question must always bear upon nursing, directly or indirectly.**

**If an answer is required by letter a fee of half-a-crown must be enclosed with the note containing the inquiry.**

*Residential Home.*

(181) Can you tell me of a home in the suburbs of Birmingham where nurses would find good accommodation between their cases?—*F. W. W.*

There are no public nurses' hostels in Birmingham, and you will have to depend on private houses for accommodation. Perhaps some nurse could recommend one from personal knowledge.

*Free Widwifery Training.*

(182) Are there any institutions where one can receive free training in midwifery without paying fees?—*Sister.*

No. A pupil always pays for being taught either in money or by service. It is, as a rule, cheaper and easier to pay in money; but it is sometimes possible to obtain instruction in return for service in private homes. Be very careful to obtain good recommendations before engaging at a private institution.

*Jersey.*

(183) Can you tell me of any hospitals in Jersey and Sark? One of my nurses has been ordered to live there. She has had two years' training here, and is quite able to do light work.—*Matron.*

Either Mrs. Hubert's Institution for Trained Nurses, 4 Victoria Crescent, Jersey; or The Nursing Institution, St. Heliers, Jersey, might be suitable.

*Addresses.*

(184) Please give me the address of the Royal British Nurses' Association; and also that of the Registered Nurses' Society?—*Frances.*

17 Old Cavendish Street, Cavendish Square, W.; 269 Regent Street, W.

*Completion of Training.*

(185) Having completed two years' hospital training, I am anxious to know if there is any institution which would give me a three years' certificate for a third year's service.—*F. E. F.*

There are a number of special hospitals which would give you an extra year's training; but, before entering into their employ, if you contemplate Poor-law work, you must ascertain from the Local Government Board, Whitehall, S.W., if the certificate you now hold and the one you propose to acquire will be accepted by that Board as equivalent to a three years' certificate. The Victoria Hospital for Diseases of the Chest, London, E., has adopted an arrangement for giving supplementary training. Write to the Matron.

*Training.*

(186) I have the chance of getting into a hospital for diseases of the chest as probationer. Will the training offered there be as useful to me as that given by a general hospital?—*Nita C.*

Unless the institution you mention offers you a three years' training, of which one year at least is spent in the wards of a general hospital, you would be well advised to seek instruction elsewhere. Nurses must be trained in a general hospital, and they must hold a three years' certificate in order to keep in line with modern requirements. A divided period of training disqualifies a nurse for some desirable posts.

*Diseases and Nursing of Children.*

(187) I should be greatly obliged if you would tell me of (1) an inexpensive book on the diseases and nursing of children; (2) one on ward management; (3) one on urine testing; (4) and one on dispensing.—*Devon.*

You can obtain from the Scientific Press (1) "Home Nursing of Sick Children," price 1s.; (2) "Hospital Sisters and their Duties," price 2s. 6d.; (3) This subject is dealt with in the ordinary nursing manuals. (4) "Notes on Pharmacy and Dispensing for Nurses," price 1s.;

*L.O.S.*

(188) Will you kindly tell a country nurse which is the best book to study for the L.O.S.?—*J. L.*

Haultain's "Practical Handbook of Midwifery" (The Scientific Press) is highly recommended.

*Soiled Bandages.*

(189) Can you tell me where the best boxes for soiled bandages and dressings can be procured suitable for a large accident hospital?—*Ireland.*

Large zinc or tin cylinder boxes with covers and handles are used by some hospitals. They are tight and easily cleaned. These things, however, should be removed from a ward at once.

*Monthly Training.*

(190) Can I possibly learn monthly nursing from a book? I am a widow with one boy, and cannot afford much time or money to be trained.—*Pattie.*

You will find it the truest economy to be properly trained; and you would not fully understand the instructions in the "Practical Handbook of Midwifery" (by Haultain, Scientific Press) without practical teaching. As a widow you could certainly claim help to enable you to become self-supporting from some of the charitable agencies. See Burdett's "Hospitals and Charities" for lists of societies.

**Standard Books of Reference.**

"The Nursing Profession: How and Where to Train." 2s. net; post free, 2s. 4d.
"The Nurses' Dictionary of Medical Terms." 2s.
"Burdett's Series of Nursing Text-Books." 1s. each.
"A Handbook for Nurses." (Illustrated.) 5s.
"Nursing: Its Theory and Practice." New Edition. 3s. 6d.
"Helps in Sickness and to Health." Fifteenth Thousand.
"The Physiological Feeding of Infants." 1s.
"The Physiological Nursery Chart." 1s.; post free, 1s. 3d.
"Hospital Expenditure: The Commissariat." 2s. 6d.

All these are published by THE SCIENTIFIC PRESS, Ltd., and may be obtained through any bookseller or direct from the publishers, 28 and 29 Southampton Street, London, W.C.

### Travel Notes.

By Our Travelling Correspondent.

### LXV. SIX MONTHS IN ROME.

### The Befána.

Among minor sights of interest is the popular festival called the Befána, celebrated on the eve of the Epiphany, but I have never heard a reliable explanation of its origin. It seems to consist of an immense noise produced from horrible tin trumpets. The Piazza Navona is its home, and here we found rows of booths containing mostly toys, tin trumpets, and cakes, though in some you could purchase fearsome old boots and clothes, &c. . . . Crowds paraded the Piazza performing on drums and trumpets: the noise was appalling, especially when some sportive soul walked behind you and blew a sudden blast in your ear. I was forcibly reminded of the Befána when I had unwarily gone into the Strand on Mafeking Day to a matinée of Duse's. The patriots' behaviour was much like that of my Italian friends. In Rome I retired from the festive scene at 7 P.M. I shudder to think what the Navona could have been like at midnight. The carabinieri, genial souls, seemed to observe the scene with an eye of paternal enjoyment. I was aroused at 1.30 A.M. by a festive fiend, who proceeded down our street enlivening the night watches with a prolonged bellow.

### The Convent of the Cappucini.

In the church itself is a Guido, St. Michael trampling upon the Devil; but it does not give one the same pleasure as Raffaelle's treatment of the subject, though it is undoubtedly fine. It is, however, in the cemetery of the Cappucini that most people find the absorbing and rather morbid interest.

We descended a few steps and were in a veritable charnel house, composed of several small chapels: round the walls were piled thousands of bones. These bones are so arranged as to leave niches in each of which was a skeleton in the full Cappucini habit. Some stood, some sat, some lay down: they were not all skeletons; some were clothed in dry parchment-like flesh. The earth in which they had been buried was brought from the Holy Land and appears to have some aromatic properties which arrests corruption, and these poor remnants of mortality are simply very dry bodies somewhat resembling mummies. As the space in the sacred earth is limited, when a fresh death takes place the last buried monk is disinterred to make room for the new comer, and is placed in one of the niches. In the chapels are piles of skulls round the altars, and the smaller bones are arranged in patterns on the walls and ceilings and (still more grisly) strung together to form chandeliers. The entire effect with the dried figures glaring out of their niches was more gruesome than I can describe. Let me give you Hans Andersen's impressions of the place as a child. I can only quote a few lines of his interesting account.

"There was fastened on the door a little cross of metal; it was fastened about the middle of the door and I could just reach it with my hand. Always when our mothers had passed by with us, they had lifted us up that we might kiss the holy sign. . . . The Capuchin monk Fra Martino was my mother's confessor 'Thou art really a bright youth,' said he, 'thou shalt now see the dead.' . . . We descended and now I saw round about me skulls upon skulls, so placed one upon another that they formed walls and several chapels. In these were regular niches in which were seated perfect skeletons enveloped in their brown cowls, their cords round their waists, and with a breviary or withered bunch of flowers in their hands. Altars, chandeliers, bas reliefs of human joints, horrible, and tasteless as the whole idea. I clung fast to the monk who whispered a prayer and then said, 'Here also I shall sometime sleep: wilt thou thus visit me?' I answered not a word but looked horrified at him and then round upon the strange grisly assembly. It was foolish to take me a child into this place. . . . I did not feel myself easy again until I came into his little cell, where the beautiful yellow oranges almost hung in at the window."

Some years later Hans Andersen again visits the awesome place: it was on the festival of All Saints, when the vaults are lighted up.

"All the monks sang masses for the dead, and I, with two other boys, swung the incense-breathing censer before the great altar of skulls. They had placed lights in the chandeliers made of bones, new garlands round the brows of the skeleton monks, and fresh bouquets in their hands. Many as usual thronged in: they all knelt, and the singers intoned the solemn Miserere. I gazed long on the pale yellow skulls and the fumes of the incense which wavered in strange shapes between me and them, and everything began to swim round before my eyes."

One must say it is a grim and horrible sight and not at all calculated to make one think happily of death and resurrection. It surely must have been a very morbid imagination that conceived so fantastic and repugnant an idea.

### The Presepio at the Ara Cœli.

A Presepio is what the French call a 'crêche' and is really a representation of the manger at Bethlehem displayed in the various churches before Christmas. That at the Ara Cœli is the best because it is the home of the sacred Bambino, a figure representing our Lord as an Infant. This Ara Cœli image is supposed to possess the power of healing the sick: it makes processions in a carriage of its own, and is always shown to the waiting crowds on the steps at Epiphany.

We found the representation very curious. The Blessed Virgin with the Bambino, Joseph a little in the background, the ox and the ass with sundry figures of dusky-complexioned shepherds, and above wreaths of rose-coloured clouds, with multitudes of angels executed in flat painted cardboard.

On the opposite side a platform on which children of various sizes were reciting by turns. Some of them were very tiny, and their courage failed them before they could utter a word; some gesticulated and turned their eyes up in a truly dramatic manner. The crowd was of varying cleanliness. I was close to a very handsome but dreadfully dirty contadino, who whenever I moved squeezed after me, intent on keeping behind some one shorter than himself. The smell of that crowd surpassed anything I have ever endured: at first resignation seemed impossible, but an occasional blessed whiff of incense mitigated the rigours of the situation.

We must now leave Rome for a little time lest those of our readers whose minds are not set towards the Eternal City should be bored. We will return there again after a short interval.

### TRAVEL NOTES AND QUERIES.

Rules in Regard to Correspondence for this Section.—All questioners must use a pseudonym for publication, but the communication must also bear the writer's own name and address as well, which will be regarded as confidential. All such communications to be addressed "Travel Editor, 'Nursing Mirror,' 28 Southampton Street, Strand." No charge will be made for inserting and answering questions in the inquiry column, and all will be answered in rotation as space permits. If an answer by letter is required, a stamped and addressed envelope must be enclosed, together with 2s. 6d., which fee will be devoted to the objects of the "'Hospital' Convalescent Fund." Any inquiries reaching the office after Monday cannot be answered in "The Mirror" of the current week."

The South of Europe for Rheumatism (Stella).—There is a great choice of places in France. First of all Dax, a season all the year round; Canterels in the Pyrenees, season May to September; Bagnères de Bigorre, June to September; Aix-les-Bains in Savoy, April to October; and La Bourboule in Auvergne, May to September. These waters are varied, and you must take medical advice as to your selection. Contrexéville is very good for gouty rheumatism.

Bruges for Cheapness (Palermo).—This is a frequent question in one form or another. Yes, it is still cheap, but not so much so as it was twenty years ago. You could get a nice little apartment suitable to your needs in a quiet street for £40. Living is much the same as in England, fruit and vegetables cheaper, grocery dearer, but the real gain is in the less ostentatious way you can live. A *femme du ménage* will be all you need for a servant, whose wages need not exceed £18 to £20 a year. There will be her keep (but they are not great eaters), and her laundry expenses.

Boarding in Paris (March).—Write to Miss Macrae, 226 Boulevard Raspail, or to Madame Mansfield, 157 Faubourg St. Honoré, also Miss Willoughby, 4 Rue des Pyramids. You must not think of advertising. Never go in Paris anywhere but where you have some recommendation. All these ladies' terms are about the same—about 35 to 45 francs per week, according to room. Thank you for your kind remarks about the Marseilles tour; I am so glad it was what you wanted.

